# AVP modulation of the vestibular nucleus via V1b receptors potentially contributes to the development of motion sickness in rat

**DOI:** 10.1186/s13041-015-0175-1

**Published:** 2015-12-12

**Authors:** Li-Hua Xu, Guan-Rong Tang, Juan-Juan Yang, Hong-Xia Liu, Jian-Cheng Li, Zheng-Lin Jiang

**Affiliations:** Department of Neurophysiology and Neuropharmacology, Institute of Nautical Medicine and Co-innovation Center of Neuroregeneration, Nantong University, 9 Seyuan Road, Chongchuan District, Nantong, Jiangsu 226019 China; Present Address: Department of Nursing, Jiangsu Provincial Xuzhou Pharmaceutical Vocational College, Xuzhou, Jiangsu 221116 China

**Keywords:** Arginine vasopressin (AVP), V1b receptor, Rotatory stimulus, Motion sickness, Conditioned taste aversion, Vestibular nucleus, Paraventricular nucleus

## Abstract

**Background:**

Arginine vasopressin (AVP) is considered to be an etiologic hormone in motion sickness (MS). The present study was designed to investigate whether individual differences in AVP expression in the paraventricular nucleus (PVN) and in modulation on the vestibular nucleus (VN) are involved in MS. Systemic application or microinjection of AVP into rat VN and rotatory stimulus were used to induce conditioned taste aversion (CTA) to 0.15 % saccharin sodium solution as a model of MS.

**Results:**

Intra-VN use of SSR149415, an antagonist of V1b receptors (V1bRs), blunted CTA. AVP inhibited Ca^2+^ influxes through L-type Ca^2+^ channels and NMDA receptor channels in cultured VN neurones, but antagonised by SSR149415. More AVP and V1bRs were expressed respectively in the PVN and VN after rotatory stimulus, especially in rats susceptible to MS. In the VN, AVP content was low, the AVP mRNA was less expressed, a few AVP-positive fibres were sparsely distributed, and fewer AVP/synaptophysin-positive terminals were identified. Almost no fluoro-ruby-labelled AVP-positive neurones in the PVN were found with retrograde tracing from the VN. SNP analysis of the reported 9 sites of the AVP gene showed significant difference between the groups susceptible and insusceptible to MS at the site rs105235842 in the allele frequencies and genotypes. However, there was not any difference between these two groups in the SNP of the reported 38 sites of V1bR gene.

**Conclusions:**

AVP, through its modulatory, possibly humoral action on the VN neurones via the mediation of V1bR, may contribute to the development of motion sickness in rats; AVP gene polymorphisms may contribute to the individual difference in the responsive expression of AVP in the PVN; and higher expressions of AVP in the PVN and V1bRs in the VN may contribute to the development of motion sickness in rats after vestibular stimulation.

## Background

Motion sickness is induced when humans receive an abnormal vestibular and/or visual stimulation while they are in actual or virtual motion. It is commonly accepted that central neuronal mechanisms are involved in the development of motion sickness, as explained by the sensory conflict theory [1–4], but the exact underlying mechanisms are still unknown. In the last decade of the 20th century, it was reported that arginine vasopressin (AVP) V1 receptor antagonists were effective in the prevention of experimental motion sickness in squirrel monkeys [5, 6]. Moreover, plasma AVP levels markedly increase during motion sickness [7–9], and intravenous or intracerebroventricular perfusion of AVP could cause nausea or vomiting in humans or in experimental animals [8–13]. Accordingly, AVP has been considered to be an etiologic hormone in motion sickness [5, 8]. Kim et al. proposed that the central, but not peripheral, actions of AVP might contribute to nausea and slow wave disruption induced by circular vection [8]. The study by Carpenter et al. suggests that the emetogenic target of peptides (including AVP) that induced emesis in dogs may be in the area postrema [10]. However, the exact mechanism underlying the etiologic effect of AVP in motion sickness in the central nervous system remains unclear [8, 9, 11].

The results from a study by Abe et al. suggest that the medial vestibular nucleus, the nucleus of the solitary tract, the area postrema, and the paraventricular nucleus (PVN) of the hypothalamus may be involved in the induction of motion sickness by hypergravity in musk shrew (Suncus murinus) [14]. It was found that there are projections from the vestibular nucleus (VN) to the PVN, and this pathway is likely polysynaptic [15, 16]. In addition, the responses of PVN neurones induced by vestibular stimuli also suggest a pathway from the VN to the PVN [17]. Vestibular stimuli will increase the expression of AVP in the PVN [18] and the secretion of AVP into the blood [7–9]. However, it is unclear whether vestibular stimulus will strengthen the vasopressinergic efferent activity of PVN. If the vestibular stimuli increase the activity of PVN vasopressinergic efferent neurones, AVP will likely induce vomiting through those efferent pathways because there are efferent innervations from the PVN to the emesis centres [19, 20]. On the other hand, Kubo et al. found reciprocal connections between the vestibular nuclei and the hypothalamus in their experiments, suggesting a potential vasopressinergic pathway from the PVN to the VN [21]. The PVN, as one of the most important autonomic control centres in the brain, may represent a unique central site at which multiple input signals can be assessed and integrated such that a complex multifactorial autonomic output can be generated [22]. The PVN, through its vasopressinergic efferent influence on neurones in the VN we supposed, might thus play an important role in the autonomic responses to motion sickness-provoking stimuli, or alternatively, AVP, acting as an etiologic hormone in motion sickness [5, 8] through its humoral modulation on neurones in the VN, might influence the development of motion sickness.

Rats do not develop the symptoms of nausea and vomiting because they lack the emetic reflex. However, rats will produce conditioned taste aversion (CTA) after a vestibular stimulus, which, as a substitute, has been used as a behavioural index of motion sickness in rats [23–27]. In the present study, we used CTA as an index of motion sickness in rats to investigate whether AVP modulation on neurones in the VN is involved in the induction of motion sickness by rotatory stimulus and whether the V1b receptors (V1bRs) of AVP in the VN mediate the motion sickness-provoking effect of AVP.

## Methods

### Animals and chemicals

Sprague–Dawley rats (200–220 g body weight) of both genders and pregnant (16 d) rats were obtained from the Experimental Animal Center of Nantong University, Nantong, China. Both genders of rats were equally allocated to each group. All procedures used in this study were in accordance with our institutional guidelines, which comply with the international rules and policies and were approved by the Animal Care and Use Committee of Nantong University, Nantong, China.

Common inorganic salts were purchased in China and culture mediums and Fluo 4-AM were obtained from Invitrogen Corporation (Carlsbad, USA). The V1bR antagonist SSR149415 was purchased from Axon Medchem (Groningen, Netherlands). Cytosine β-D-arabinofuranoside, sodium dodecylsulphate, AVP and other chemicals, except those indicated elsewhere, were purchased from Sigma-Aldrich Corporation (Saint Louis, USA).

### Motion sickness-inductive rotatory stimulus

The rotatory stimulator for use in small animals was manufactured according to the report by Crampton and Lucot [28]. Briefly, two clear lucite boxes are hung over a frame that turns around a horizontal axis. An electric motor drives the device, and a transducer (DZB60J 1.5 kW, Electric Fuling, Wenling, Zhejiang Province, China) controls its time and rate of acceleration or deceleration, velocity, rotation duration, direction (clockwise and counter clockwise) and brake. Rats can move freely in these boxes with unrestricted vision of the laboratory environment [27].

For the induction of motion sickness, rats were rotated in the stimulator in an alternate acceleration and deceleration mode. The acceleration rate was 16 °/s^2^ for a duration of 7.5 s with a maximal velocity of 120 °/s, and the deceleration rate was 48 °/s^2^ for a duration of 2.5 s. The clockwise and counter clockwise rotations were alternately repeated for 120 min. As suggested by the literature [23–27], conditioned taste aversion served as the index (“being sick”) of motion sickness. Before rotatory stimulus, 0.15 % saccharin sodium solution (SSS) was supplied for the rats to drink for 45 min after they had been deprived of water for 24 h and had become familiar with this novel fluid one day before water deprivation through free access to SSS in addition to tap water for another 24 h. After a 24-h rest with free access to tap water, the rats were subjected to rotatory stimulus, and after another 24-h water deprivation, 0.15 % SSS was then supplied for 45 min and the intake volume was measured. The rats with intake volume decreases of less than 15 % were considered to be insusceptible to motion sickness, in contrast, the rats with intake volume decreases greater than 15 % were considered to be susceptible.

For the experimental control, before and after the rotatory stimulus, control rats of both genders were supplied with tap water instead of 0.15 % SSS for the same durations of 45 min after 24-h water deprivations. To avoid the influence of water deprivation on AVP secretion, the rats used for the above measures of taste aversion were not included in the following determinations of AVP expression.

### Intracerebral microinjection

The rats were placed in a stereotaxic frame (Stoelting Co., Wood Dale, USA) under an anaesthesia of 10 % chloral hydrate (400 mg/kg, i.p.). Over a 15-min period, 0.1 μl of AVP and/or V1bR antagonist SSR149415 dissolved in normal saline was slowly injected into the vestibular nucleus through a 1-μl microsyringe (Hamilton Company, Reno, USA) with the coordinates of VN: 12.3 mm posterior to the bregma, 1.2 mm lateral to the midline, and at a depth of 7.4 mm under the meninges [29] and with a retention time of 5 min. Another side of the VN was successively microinjected in the same way. The vehicle rats were injected with an equal volume of normal saline.

To observe whether local use of AVP in VN could induce CTA, before intracerebral microinjection, a 0.15 % saccharin sodium solution was supplied for the rats to drink for 45 min after they had been deprived of water for 24 h and had become familiar with this solution one day before water deprivation through free access to SSS in addition to tap water for another 24 h. After a 24-h rest with free access to tap water, a bilateral microinjection of AVP (10 ng or 30 ng for each side), SSR149415 (5.7 ng or 17.2 ng for each side) or their combination into the VN was performed, the rats were deprived of water for another 24-h period and were then given another 45-min of free access to SSS. The intake volume of SSS was measured, and changes after the AVP microinjection were calculated.

To investigate whether the AVP V1bR antagonist also influences the induction of CTA after rotatory stimulation, SSR149415 (5.7 ng for each side) was microinjected bilaterally into the VN. AVP microinjection was substituted with a 2-h rotatory stimulus; the other procedures were the same as those in the AVP microinjection experiment.

To observe whether local use of the AVP V1bR antagonist in VN blocks the motion sickness-inductive effect by systemic use of AVP, rotatory stimulus was substituted with an intraperitoneal injection of AVP (100 μg/kg) to induce CTA; the other procedures were the same as those of above SSR149415 (5.7 ng for each side) microinjection experiment and rotatory stimulus-induced CTA.

To retrogradely trace AVP fibres that potentially terminate in the VN, a retrograde tracer Fluoro-ruby (10 %, 0.15 μl) was microinjected into the right side of the VN for 3 min, and the micropipette was left in place for 5 min before withdrawal to minimise the leaking of the tracer. After 8 days, the animals received an anaesthesia with 10 % chloral hydrate followed with the slicing of the rat brain tissue, immunohistochemistry, and microscopic observations.

### Nissl staining and immunohistochemistry in brain tissue

The rats were anaesthetised with 10 % chloral hydrate (400 mg/kg, i.p.) and perfused with 200 ml of saline and then with 4 % paraformaldehyde in 0.1 M phosphate buffered saline (PBS, pH 7.4). The rat brains were then removed and post-fixed for 24 h in the same fixative. The post-fixed brains were dry-protected in PBS containing 30 % sucrose. For Nissl staining, the brains were sectioned into slices of 30 μm in thickness with a cryostat slicer (CM1900, Leica, Bensheim, Germany). For immunofluorescent staining, the brains were sectioned coronally with a thickness of 5 μm.

For Nissl staining, the brain sections were mounted with neutral balata and blotted onto slides before being processed through different baths in the following order (and times): chloroform (30 min), acetone (15 min), 100 % ethanol (30 s), 95 % ethanol (30 s), 70 % ethanol (30 s), distilled water (30 s, twice), cresyl violet (20 min), distilled water (30 s, three times), 70 % ethanol (1 min), 95 % ethanol (1 min), 100 % ethanol (1 min), chloroform (5 min), differentiator (95 % ethanol, added glacial acetic acid till pH was 4.1) (6 min), 95 % ethanol (2 min), 100 % ethanol (3 min, twice), xylene (2 min), and xylene (3 min, twice); they were then covered with a coverslip.

For immunofluorescent staining, the brain sections were blocked and permeabilised with 0.3 % Triton X-100 and 8 % normal donkey serum (Jackson, West Grove, USA) in PBS. The sections were then incubated with 0.3 % Triton X-100 and 4 % normal donkey serum in PBS containing primary antibodies in the following dilutions: rabbit anti-AVP (EMD Millipore Corporation, Billerica, USA) 1:2000 and mouse anti-synaptophysin (EMD Millipore Corporation, Billerica, USA) 1:2000 overnight at 4 °C. The secondary antibodies used were donkey anti-rabbit Alexa-488 and donkey anti-mouse Alexa-594 or donkey anti-rabbit Alexa-647 (1:1000, Jackson, West Grove, USA) for 2 h at room temperature. The sections were imaged using a confocal microscope (TCS SP8, Leica Microsystems, Wetzlar, Germany).

### Enzyme-linked immunosorbent assay (ELISA)

Two hours after rotatory stimulus, the rat brains were harvested under anaesthesia (10 % chloral hydrate, 400 mg/kg, i.p.), and were sliced in the ice-cold artificial cerebral-spinal fluid to a thickness of 350 μm with a vibratome (Campden Instruments, UK). According to the location of the VN and PVN in the brain atlas [29], the tissues of these two areas were cautiously isolated for the following measurements including ELISA, real-time quantitative PCR (qRT-PCR) and western-blot analysis.

The content of AVP in the VN and PVN was measured with ELISA. The tissues of the VN and PVN were isolated and stored at −80 °C until use. The brain tissues were homogenised and centrifuged at 4 °C and 14,000 rpm for 30 min. The supernatants were transferred to new eppendorf tubes and assayed in duplicate using AVP assay kits (R&D Systems, Minneapolis, USA) according to the manufacturer’s guidelines.

### Real-time quantitative PCR

The tissues isolated from the rat brains were homogenised in Trizol reagent (Invitrogen Corporation, Carlsbad, USA), and the total RNA was purified. Potential contamination of DNA was removed using DNase, and 1 μg of RNA was used for first strand complementary DNA (cDNA) synthesis by retrotranscription using oligodT primers and the PrimeScript RT Reagent Kit (Tiangen Biotech Co., Ltd, Beijing, China) according to the manufacturer's instructions. qRT-PCR reactions were carried out in a Rotor-Gene PCR machine (RG-3000A, Corbett Research Proprietary Limited, New South Wales, Australia), and the detailed procedures were as follows: an initial denaturation step at 95 °C for 5 s, followed by 40 cycles of a 95 °C denaturation for 5 s, 60 °C annealing for 20 s, and 72 °C extension for 30 s. The amounts of cDNA per sample were determined using a SYBR Premix Ex Taq™ kit (Roche, Basel, Switzerland). The progression of the PCR reactions was assessed by changes in the SYBR green dye fluorescence attached to double stranded DNA. All values were normalised to the housekeeping gene β-actin. The primers used for qRT-PCR were: AVP forward: 5′-CTCGGGAGCAGAGCAACG-3′, reverse: 5′-GGGGCGATGGCTCAGTAG-3′; V1bR forward: 5′-GGGCAGATTTCTACTTT-3′, reverse: 5′-CTGGGTCTTGACTTTC-3′; β-actin: forward: 5′-TCTACATGTTCCAGTATGACTC-3′, and reverse: 5′-ACTCCACGACATACTCAGCACC-3′.

### Western-blot analysis

The brain tissues were lysed in 1 ml of tissue lysis solution containing phenylmethanesulfonyl fluoride (Beyotime, Nantong, China), and then centrifuged at 13,400 × g for 15 min at 4 °C. The protein content of the supernatant was determined spectrophotometrically using the bicinchoninic acid method. Equal amounts of protein (40 μg per lane) from each sample were loaded on a 10 % sodium dodecyl sulphate-polyacrylamide gel, electrophoresed, and transferred onto the polyvinyledene difluoride membranes (Millipore, Massachusetts, America). These membranes were blocked in 5 % non-fat milk in Tris-buffered saline containing 0.1 % Tween-20 for 2 h at room temperature and were incubated overnight at 4 °C with primary antibodies (diluted in Tris-buffered saline containing 0.1 % Tween-20 with 5 % non-fat milk), V1bR (1:500, Lifespan, BioSciences, Inc., Seattle, USA) and GAPDH (1:6000, Sigma-Aldrich Corporation, Saint Louis, USA). After incubating the blots with rabbit anti-goat IgG (H + L)-HRP (1:10000, Bioworld, Nanjing, China) for 2 h at room temperature, the immunoreactive bands were visualised by the enhanced chemiluminescence (Prierce Biotechnology, Rockford, USA) and exposed to X-ray film (Eastman Kodak Company, Rochester, USA). The images were captured using an EC3 Imaging System, and the protein levels were quantified using Image-Pro Plus. The same samples were analysed repeatedly for a minimum of two times.

### Cell culture

Primary vestibular nuclei cultures were established from the embryonic (16 d) rats. Foetuses were removed from the uterus and placed in sterile ice-cold Hank's balanced salt solution (Gibco, Carlsbad, USA). The foetal rat brains were removed with the aid of a dissecting microscope by dissection of the skull using fine-tipped watchmaker’s forceps. The vestibular nuclei were carefully dissected from the brain stem, and the blood vessels, meninges and other brain tissues were removed. The tissue matrix was loosened by treatment with 0.25 % trypsin (Life Technologies, Carlsbad, USA) for 6 min at 37 °C in a shaking water bath.

After mechanical trituration and centrifugation, the resulting cell pellet was resuspended in Dulbecco's Minimum Essential Medium containing 10 % foetal bovine serum, 1 % GlutaMAX, and 1 % penicillin-streptomycin (all from Life Technologies). The cells were seeded at a density of 1 × 10^5^ cells per well in 24-well poly-D-lysine-coated plates. Four hours later, the medium was changed with neurobasal medium (Life Technologies) supplemented with 2 % B27 (Life Technologies), 1 % GlutaMAX and 1 % penicillin-streptomycin. The cultures were maintained by a 50 % media exchange every 3 days for at least 12 days. The day of plating was counted as day-in-vitro 0. On day-in-vitro 3, cytosine-β-D-arabinofuranoside (5 μM, Sigma-Aldrich Corporation, Saint Louis, USA) was added to suppress the proliferation of glial cells for 3 days followed by a return to regular neurobasal medium. The cell plates were maintained at 37 °C in a humidified 5 % CO_2_ incubator.

### Intracellular Ca^2+^ imaging

For the imaging of the intracellular free Ca^2+^, Fluo 4-AM (the acetoxymethyl-ester form of Fluo 4) was used as a fluorescent Ca^2+^ indicator. The cultured vestibular nuclei neurones on day-in-vitro 12 were loaded with 5 μM Fluo 4-AM in extracellular solution for 45 min at 37 °C. After being washed three times with normal extracellular solution, the neurones were incubated at 37 °C for another 30 min to complete the deesterification of Fluo 4-AM. The intensity of fluorescence with the excitation wavelength at 485 nm and an emission wavelength at 525 nm was recorded every 10 s using a laser scanning confocal microscope (TCS SP8, Leica Microsystems). All image data were collected and analysed with the Leica Control software of the microscope. The increase of intracellular free Ca^2+^ was determined according to the following equation: Ca^2+^ influx (%) = (F_525_ − F_base, 525_) / F_base, 525_ × 100, where F_525_ is the fluorescence intensity measured after each treatment and F_base, 525_ is the basal fluorescence intensity. A high extracellular K^+^ solution containing (in mM): 98 NaCl, 50 KCl, 10 HEPES, 3 CaCl_2_°2H_2_O, 2 MgCl_2_°6H_2_O, and 8 glucose was used for the induction of Ca^2+^ influx through the L-type Ca^2+^ channels. A NMDA solution containing (in mM): 98 NaCl, 1 NMDA, 10 HEPES, 3 CaCl_2_°2H_2_O, 2 MgCl_2_°6H_2_O, and 8 glucose was used for the induction of Ca^2+^ influx through the NMDA receptor channels.

### The analysis of single nucleotide polymorphism (SNP) of the AVP and V1bR genes

Whole brain tissues of rats were isolated under anaesthesia with 10 % chloral hydrate (400 mg/kg, i.p.). Genomic DNA was extracted from the rat brains using an AxyPrep Genomic DNA Mini Preparation Kit (Axygen Scientific, Inc., Union City, USA) following the manufacturer’s protocol and checked for concentration and purity using a NanoDrop 2000 Biophotometer (Thermo Scientific, Milford, USA). Primers for the AVP and V1bR genes (NC_005102.2 and NC_005112.2) for SNP analysis were designed using Primer Premier 5.0 software (PREMIER Biosoft, Palo Alto, USA), with technology support by Shanghai R & S Co., Ltd. According to the reported 9 sites of AVP gene SNP and the 38 sites of V1bR gene SNP listed in NCBI through March 2011 (Table 1, http://www.ncbi.nlm.nih.gov/pubmed/), we designed primer pairs of 5 fragments in the AVP gene and 13 fragments in the V1bR gene, as shown in Table 2.

**Table 1:** List of AVP and V1b receptor gene SNP sites

AVP gene SNP sites	V1b receptor gene SNP sites
rs8175020, rs105049089, rs105235842, rs105342065, rs105406202, rs106248204, rs106280676, rs107234383, rs197835086	rs8174376, rs8174377, rs104934522, rs104972087, rs105131484, rs105281630, rs105361773, rs105397955, rs105426072, rs105440759, rs105623482, rs105762638, rs105845580, rs105862475, rs106031473, rs106081862, rs106100029, rs106125862, rs106220176, rs106257682, rs106366335, rs106372777, rs106473080, rs106531259, rs106595881, rs106658396, rs106778661, rs106795491, rs106849188, rs106924578, rs106967487, rs107136421, rs107203534, rs107214944, rs107222006, rs107252548, rs107371590, rs107406983

**Table 2:** Primer pairs of AVP and V1b receptor genes

Primer	Sequence	Product size (bp)
AVP-F1	GTCCTTCACGTTGTTTTTGCCTTA	560
AVP-R1	GTACAGCTGGCTGGGACACAA	
AVP-F2	TCTCTGAAGGAAGGGCTGTGT	444
AVP-R2	TCCTTCCCCGCAGTGTCTC	
AVP-F3	TCTCCAAAGGAACTCAGCAAA	350
AVP-R3	CTGGGTAGATGGTACGAAACTGTT	
AVP-F4	ACTCTTGATCTTTCTATCTCCACCT	377
AVP-R4	TCCCTACACATGAGCTGTCTCTTAT	
AVP-F5	GCTTCGTGTTAGTAATGTCCTTGTT	432
AVP-R5	GGCAGAGGTAGTGAGTTTGAGTTT	
AVPr1b-1-F	GCACAGAGACTGAAAGTAATTGGCT	428
AVPr1b-1-R	CTGTCTGAAAGGCGGCGG	
AVPr1b-2-F	CTCAGCCCCTCCCCTCAGTA	621
AVPr1b-2-R	ATGAGAGAGAAAGGTTTAGAGGTGG	
AVPr1b-3-F	GGCAGCCCAGCCAGTCTAC	472
AVPr1b-3-R	CCACATCTGGACACTGAAGAAAGG	
AVPr1b-4-F	AGGGCCTTGCGCTTCCTAG	266
AVPr1b-4-R	ATTCATTCAACATAGCCTTAGTGGG	
AVPr1b-5-F	AGGTATACAATGTTCTGCCTGCC	418
AVPr1b-5-R	AAGGGAAGGGCACCCAGAG	
AVPr1b-6-F	TCTACAAAGAGAGAGGCTTTCCC	335
AVPr1b-6-R	GCCTTGGCTAACATCCTTAATAG	
AVPr1b-7-F	CTCGGTGAGAGCGAAGAATTTC	363
AVPr1b-7-R	GGTTCTCAACCTTAGTGCCACG	
AVPr1b-8-F	GTTGAGGGTCACCACAACACG	431
AVPr1b-8-R	TCAGGCCCAAAGCAAGAGATC	
AVPr1b-9-F	GATGGACATGAACCTCTGACCTC	697
AVPr1b-9-R	TGCTGAGTTTCTAAAAAGCGAAG	
AVPr1b-10-F	ATGACCTGACACAACGTGGAAG	454
AVPr1b-10-R	TGGAGATAATTAAGAGCCATCGC	
AVPr1b-11-F	CAATCGTGGTGCCCCAAAC	296
AVPr1b-11-R	ATGGTTGTGAGCCACCATGTG	
AVPr1b-12-F	CCAAATGGACACAACCAGAGAAG	652
AVPr1b-12-R	GGAATAGTTTGCTTCAAGCTCAGG	
AVPr1b-13-F	CTGCAACCCCTGGATCTACATG	400
AVPr1b-13-R	CATTCTGGCCTCTTCACCCTG	

To obtain higher accuracy, amplification of the AVP sequences using Taq high-fidelity DNA polymerase was performed. Semi-quantitative RT-PCR was carried out in a 50 μL reaction volume containing 10 × buffer 5 μL, MgSO_4_ (50 mM) 2 μL, dNTP (10 mM) 1 μL, and with each of the forward and reverse primers (10 μM) 1 μL, DNA 1 μL, Taq HIFI (5 U/μL, Invitrogen Corporation) 0.2 μL, and ddH_2_O added to the final volume. The PCR cycling protocol was an initial denaturation at 95 °C for 5 min, 35 cycles of 95 °C denaturation for 30 s, 57–65 °C annealing for 30 s, and 68 °C extension for 30 s. The PCR products of 3 μL were separated by electrophoresis in a 1.5 % agarose gel to verify their integrity, the others were purified using a PCR purification kit (Axygen Scientific, Inc.) following the manufacturer’s protocol. The purified products were sequenced by ABI 3730xl DNA analyser (Applied Biosystems, Carlsbad, USA) to analyse the SNP information.

### Statistical analysis

All data are presented as the mean ± s.e.m. Student’s *t*-tests of paired or unpaired observations were used, respectively, for the comparisons of two groups, paired or unpaired. One-way ANOVA was used for the comparison of three or more groups, and the Least Significant Difference test was used for post hoc comparisons between the two groups. The chi-square test was used for the comparison of rates in the SNP analysis of AVP and V1bR genes. Differences were considered statistically significant at a level of *P* < 0.05.

## Results

### Induction of CTA and mediation through V1bR in VN

**Fig. 1:**
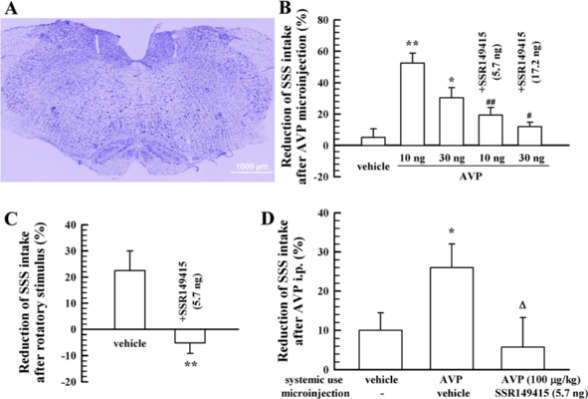
Induction of CTA by a rotatory stimulus or intra-vestibular nuclei microinjection of AVP and the effects of the V1bR antagonist (*n* = 10). **a**, indication of the microinjection area in the VN (Nissl staining). **b**, induction of CTA by AVP microinjection into the VN and the blocking effect of V1bR antagonist SSR149415. **c**, induction of CTA after rotatory stimulus and the blocking effect of SSR149415 microinjection into the VN. **d**, induction of CTA after AVP intraperitoneal injection (i.p.) and the blocking effect of SSR149415 microinjection into the VN. ^*^ *P* < 0.05, ^**^ *P* < 0.01, vs. vehicle; ^#^ *P* < 0.05, ^##^ *P* < 0.01 vs. AVP 10 ng or 30 ng groups, respectively; ^Δ^ *P* < 0.05, vs. AVP (i.p.) used alone

Nissl staining of the brain slices was performed immediately after the behavioural tests of CTA to identify whether the microinjection sites were bilaterally right in the VNs (Fig. 1a). Data from rats (about 2–3 animals for each group) with injections bilaterally asymmetrical or not right in the VNs were not included in the following results. In addition, the negative control experiments revealed that there were not any differences in the intake volume of tap water before and after different treatments including rotatory stimulus, AVP injection or use of AVP V1bR antagonist. Similar to rotatory stimulus, microinjection of AVP into the vestibular nuclei induced CTA in rats, i.e., corresponding to an intra-vestibular nuclei microinjection of 10 ng or 30 ng of AVP, the intake volume of SSS decreased compared with that before microinjection (Fig. 1b). However, when AVP V1bR antagonist SSR149415 was injected at the same time, the decrease in the intake volume of SSS elicited by AVP was significantly inhibited. In addition, to identify the involvement of V1bR in the induction of rotatory stimulation- and AVP intraperitoneal injection-induced CTA, we also observed the effect of SSR149415 injection into the vestibular nuclei. As a result, microinjection of SSR149415 into the VN also inhibited rotatory stimulus- and AVP intraperitoneal injection-induced CTA in rats (Fig. 1c and d). These results suggest that rotatory stimulation-induced motion sickness in rats is likely related to humoral modulation by AVP in the vestibular nuclei through V1bR.

### The inhibitory influence of AVP on L-type voltage-dependent Ca^2+^ channels in cultured VN neurones

**Fig. 2:**
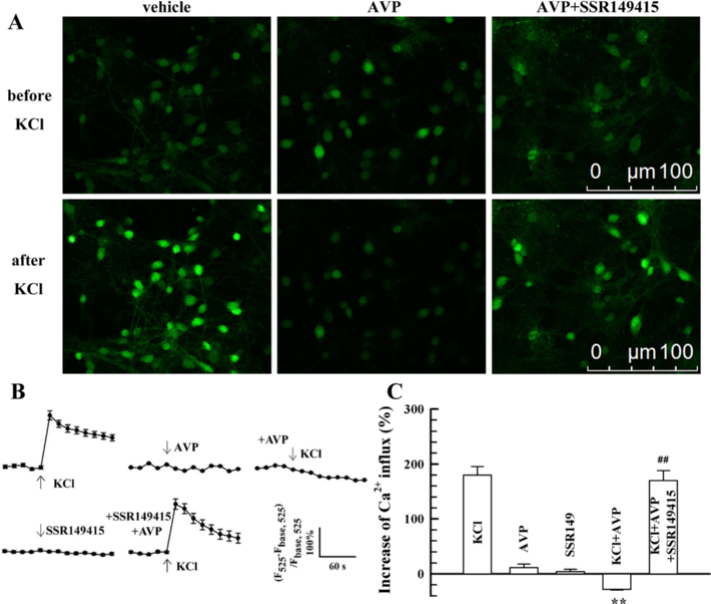
The influence of AVP on KCl-elicited Ca^2+^ influx (*n* = 20). **a**, examples of Ca^2+^ imaging. **b**, mean values of Ca^2+^ influx changes after different treatments. **c**, mean peak values of Ca^2+^ influx after different treatments. ^**^ *P* < 0.01, vs. KCl group. ^##^ *P* < 0.01, vs. KCl + AVP group

Ca^2+^ influx was enhanced prominently when KCl (50 mM) was infused into the cultured VN neurones (Fig. 2). This enhancement was inhibited by pre-incubation with 100 nM AVP in the extracellular solution for 60 min (*P* < 0.01, Fig. 2). The addition of AVP (100 nM) or V1bR antagonist SSR149415 (100 nM) alone did not elicit any notable Ca^2+^ influx. Pre-incubation of SSR149415 with AVP in the extracellular solution for 60 min removed the inhibitory effect of AVP on the KCl-elicited Ca^2+^ influx (*P* < 0.01, Fig. 2).

#### The inhibitory influence of AVP on NMDA receptors in cultured VN neurones

**Fig. 3:**
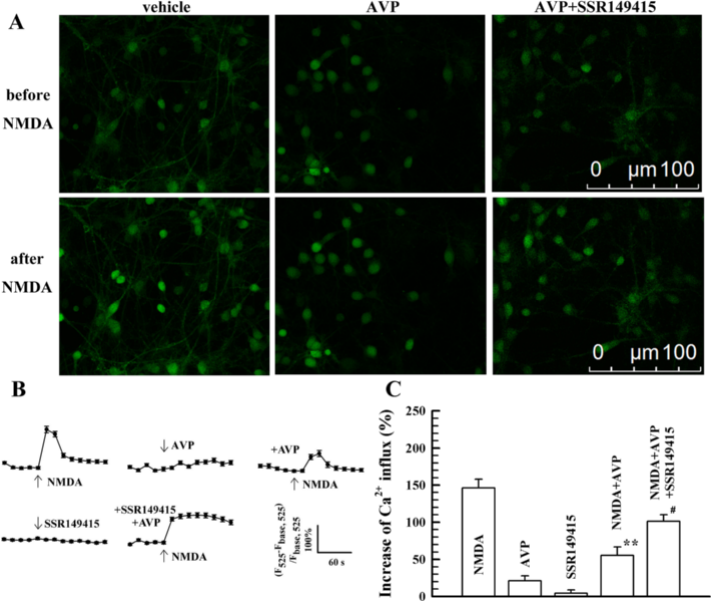
The influence of AVP on NMDA-elicited Ca^2+^ influx (*n* = 20). **a**, examples of Ca^2+^ imaging. **b**, mean values of Ca^2+^ influx changes after different treatments. **c**, mean peak values of Ca^2+^ influx after different treatments. ^**^ *P* < 0.01, vs. NMDA group. ^##^ *P* < 0.01, vs. NMDA + AVP group

Perfusion of NMDA (1 mM) to the cultured VN neurones remarkably elicited an influx of Ca^2+^ (Fig. 3). The addition of AVP (100 nM) or V1bR antagonist SSR149415 (100 nM) alone did not elicit an influx of Ca^2+^ (Fig. 3). After the pre-incubation of AVP for 60 min, the NMDA-elicited Ca^2+^ influx was significantly inhibited (*P* < 0.01, Fig. 3), but pre-incubation of SSR149415 with AVP in the extracellular solution for 60 min blunted the inhibitory effect of AVP on the NMDA-elicited Ca^2+^ influx (*P* < 0.01, Fig. 3).

#### The expression of V1bR in the VN

**Fig. 4:**
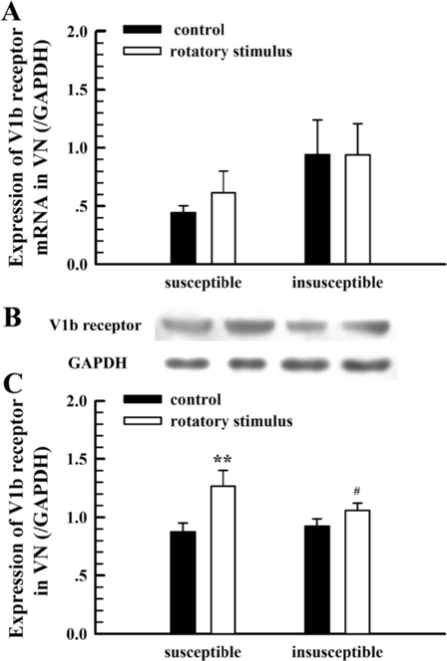
The expression of V1bR in the VN with and without rotatory stimulus. **a**, expression of V1bR mRNA (*n* = 8). **b**, examples of the V1bR protein expression. Subgroups correspond to the columns of (**a**) and (**c**). **c**, mean values of the V1bR protein expression (*n* = 6). ^**^ *P* < 0.01, vs. control; ^#^ *P* < 0.05, vs. susceptible group after rotatory stimulus

As shown in Fig. 4a, no significant changes were found in the expression of V1bR mRNA in the VN between the groups with and without rotatory stimulus. However, the expression of V1bR protein was elevated after rotatory stimulus, especially in the group susceptible to motion sickness (Fig. 4b).

#### The expression of AVP in the PVN and the distribution of AVP positive fibres and vasopressinergic nerve endings in the VN

**Fig. 5:**
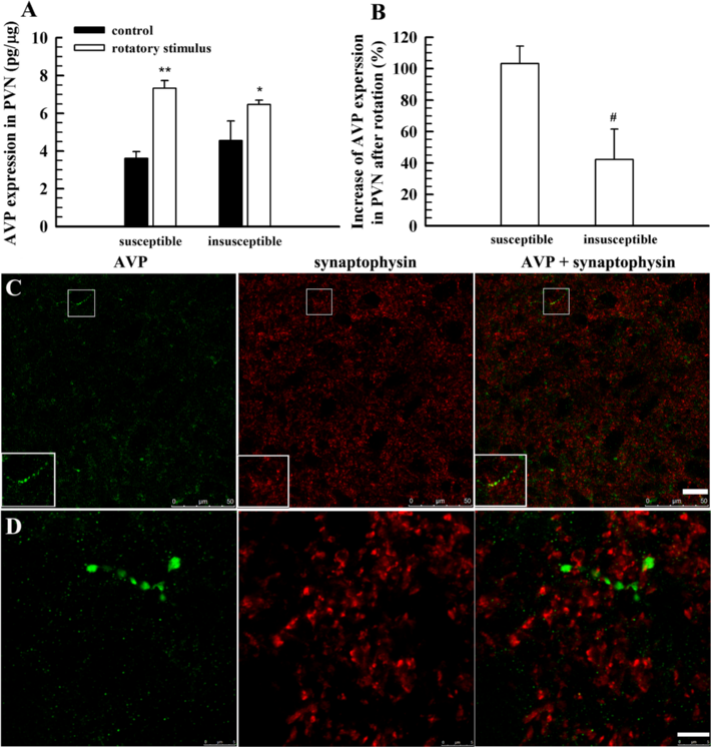
The expression of AVP in the PVN and the distribution of AVP-positive fibres in the VN. **a**, AVP expression in the PVN of susceptible and insusceptible groups with and without rotatory stimulus (*n* = 6); **b**, net increase of AVP expression in the PVN after rotatory stimulus (*n* = 6); **c**, labelling of AVP and synaptophysin; **d**, another section labelling AVP and synaptophysin and further magnification to reveal AVP fibre terminals that express synaptophysin. Four rats, two for each gender, were used in (**c**) and (**d**). ^*^ *P* < 0.05, ^**^ *P* < 0.01, vs. control. Scale bar, 20 μm for (**c**), 5 μm for (**d**)

The expression level of AVP mRNA (relative to β-actin) in the PVN was 0.7219 ± 0.1062 (*n* = 6). However, the expression level of AVP mRNA in VN was 0.0090 ± 0.0008 (*n* = 6), which was much lower than that in the PVN. It is suggested that the VN itself possibly does not synthesise AVP. Then, we measured the expression level of AVP in the PVN and VN with ELISA. The AVP level in the VN was much lower than that in the PVN and was not significantly changed after rotatory stimulation (data not shown). As shown in Fig. 5a, the expression of AVP in the PVN was elevated after rotatory stimulus, especially in rats of the group susceptible to motion sickness (Fig. 5b).

A sparse distribution of AVP-positive fibres in the VN was found using immunofluorescent staining method, but no AVP-positive cells were identified in the VN (Fig. 5c and d). In addition, we did not find a notable difference in the distribution of AVP-positive fibres in the VN between the susceptible and insusceptible groups. To find vasopressinergic nerve endings in the VN, a double labelling method was used to reveal AVP and synaptophysin, a marker of vesicles that is present in both the synaptic vesicles and the large dense-cored vesicles [30–33]. As a result, very few double labelling puncta were found (Fig. 5c and d), suggesting that there are few vasopressinergic nerve endings that terminate in the VN.

#### Retrograde tracking of AVP fibres to the VN

**Fig. 6:**
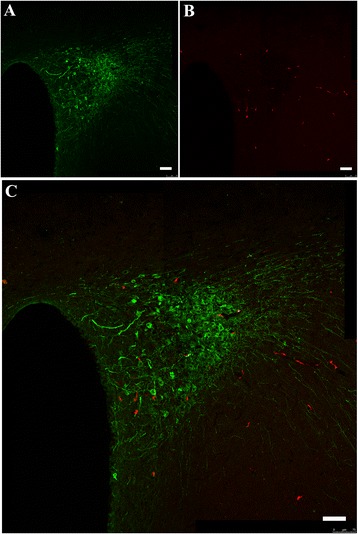
Retrograde tracking of AVP fibres from the PVN to the VN. Four rats, 2 for each gender, were used. Serial sections covering the PVN were taken from each animal for observation. **a**, AVP-positive cells in the PVN; **b**, fluoro-ruby-labelled cells through retrograde tracking from the VN; **c**, merge of (**a**) and (**b**) and further magnification. Scale bar, 75 μm

To further identify whether there are abundant vasopressinergic nerve fibre innervations in the VN originated from the PVN, we performed additional observation through retrograde tracking of AVP fibres to the VN. As shown in Fig. 6, eight days after injection of fluoro-ruby into the VN, only some small cells in the PVN without notable AVP labelling (green) were bilaterally labelled with fluoro-ruby (red), suggesting that there are not vasopressinergic nerve fibres in the PVN that directly innervate the VN neurones.

#### The difference in single nucleotide polymorphisms of the AVP and V1bR genes

Single nucleotide polymorphism analysis of the reported 9 sites in the AVP gene showed that at the site rs105235842 (functional consequence: upstream variant 2 KB, http://www.ncbi.nlm.nih.gov/snp/?term=rs105235842), a difference between the susceptible and insusceptible groups was found in the allele frequencies and genotypes (*P* < 0.01, Table 3), where single nucleotide T was the dominant allele.

**Table 3:** Allele frequencies and genotypes of rat AVP gene site (rs105235842)

Group	Number	Genotype (%)			Allele frenquency (%)	
		T/T	C/C	T/C	T	C
Susceptible to motion sickness	12	41.7 (5)	0 (0)	58.3 (7)	70.8	29.2
Iinsusceptible to motion sickness	12	100 (12)	0 (0)	0 (0)	100	0
		χ^2^ = 9.471, *P* = 0.0046			χ^2^ = 8.195, *P* = 0.0094	

In addition, at the site rs197835086 (functional consequence: intron variant, http://www.ncbi.nlm.nih.gov/snp/?term=rs197835086), a difference between the susceptible and insusceptible groups was found in the genotypes, but was statistically insignificant (*P* = 0.0617, Table 4), where single nucleotide A was the dominant allele.

**Table 4:** Allele frequencies and genotypes of rat AVP gene site (rs197835086)

Group	Number	Genotype (%)			Allele frenquency (%)	
		A/A	G/G	A/G	A	G
Susceptible to motion sickness	12	50.0 (6)	0 (0)	50.0 (6)	75.0	25.0
Insusceptible to motion sickness	12	83.3 (10)	8.3 (1)	8.3 (1)	87.5	12.5
		χ^2^ = 3.573, *P* = 0.0617			χ^2^ = 1.231, *P* = 0.4614	

SNP analysis revealed that there was not any difference between the susceptible and insusceptible groups of rats in the SNP of the reported 38 sites in the V1bR gene we investigated in the present study.

## Discussion

In the present study, we first proved that the bilateral microinjection of AVP into vestibular nuclei, similar to a rotatory stimulus, induced conditioned taste aversion in rats and that the application of SSR149415, an antagonist of AVP V1bR, blunted this CTA-inducing effect of AVP and rotatory stimulus-induced CTA, suggesting that AVP, through its action on the VN, could contribute to the development of motion sickness in rats via the mediation of V1bR. In addition, we found that AVP inhibited Ca^2+^ influxes through L-type Ca^2+^ channels and NMDA receptor channels in cultured VN neurones, and this effect was reduced in the presence of the V1bR antagonist SSR149415, indicating that AVP potentially exerts an inhibitory influence on the activity of VN neurones via V1bR. This inhibitory influence is consistent with the in vivo results reported by Podda et al. [34]. Ballesteros and Gallo found that a bilateral blockade of the lateral vestibular nucleus by tetrodotoxin microinjections could also induce CTA, which is consistent with our results [35]. Furthermore, the expression of V1bR in the VN was confirmed with qRT-PCR and western-blot analysis in the present study and had a greater expression after rotatory stimulus, especially in the rats susceptible to motion sickness. This finding is consistent with our previous results concerning the V1bR-positive cells in the VN of rat after rotatory stimulus observed using the immunofluorescence imaging method [36].

Moreover, the present study found low expressions of AVP mRNA and no AVP-positive cells in the VN, suggesting that the VN itself possibly does not have vasopressinergic cells to synthesise AVP. Thereby, the lower content of AVP found in the VN is consistent with this suggestion. However, some AVP-positive fibres and few AVP/synaptophysin-positive fibres in the VN were found in the present study. In addition, few fluoro-ruby-labelled AVP-positive neurones in the PVN were found using the retrograde tracing method. These results suggest that there are few vasopressinergic nerve endings that terminate in the VN of rats and that there are not direct innervations in the VN of AVP-positive fibres originating from the PVN. AVP existing in the fibres passing through the VN is likely the source of the low levels of AVP in the VN.

Therefore, to modulate the vestibular neurones, we suggest that AVP might come from the blood stream or from cerebrospinal fluid (CSF). The location of the vestibular nucleus that dorsally borders the floor of the fourth cerebral ventricle affords the convenience for the potential modulation of AVP from CSF [37]. Crampton and Daunton found evidence for a motion sickness agent in the CSF of cats, but whether AVP was the humoral motion sickness substance was a matter of conjecture at that time [38]. Moreover, it is reported that plasma AVP levels increase markedly during motion sickness [7–9] and that intravenous or intracerebroventricular perfusion of AVP indeed causes nausea or vomiting in humans and experimental animals [8–13]. AVP has been considered as an etiologic hormone in the development of motion sickness [5, 8]. We also found an increase in AVP levels in the blood after motion sickness-inductive rotatory stimulation in both dogs and rats [39, 40]. In the present study, our further experiment revealed that bilateral microinjection of SSR149415 into the VN blunted the CTA induced by intraperitoneal injection of AVP. Thus, combined with the present results and those from other studies [5–13], we postulate that AVP might be the candidate of motion sickness-provoking agent in the CSF or in the blood and that the VN might be one of the most important candidates targeted by AVP.

In the present study, we found that the AVP levels in the PVN were elevated after rotatory stimulus, especially in rats susceptible to motion sickness, which was consistent with other studies showing that vestibular stimulus increased the AVP-expressing cells in the PVN [18], which is likely the source of AVP secreted into blood during the development of motion sickness [7–9] or after motion sickness-inductive rotatory stimulation [39, 40]. Moreover, the expression of V1bR protein in the VN was elevated after rotatory stimulus, especially in the group susceptible to motion sickness. These results suggest that the relative high sensitivity of rats susceptible to motion sickness is likely associated with a greater expression of AVP in the PVN and increased expression of AVP V1bR in the VN. To investigate the potential mechanisms of these differences in the expression of AVP and V1bR in rats of different susceptibility to motion sickness, the SNPs of the reported 9 sites in the AVP gene and 38 sites in the V1bR gene were analysed. We found that at the SNP site rs105235842, a difference between the susceptible and insusceptible groups was found in the allele frequencies and genotypes, and at the site rs197835086, a difference between the susceptible and insusceptible groups was found in the genotypes. The SNP site rs105235842 is an upstream variant of the AVP gene (http://www.ncbi.nlm.nih.gov/snp/?term=rs105235842), and the SNP site rs197835086 is an intron variant (http://www.ncbi.nlm.nih.gov/snp/?term=rs197835086). These two variances may influence the transcription of the AVP gene and result in the difference in the expression levels of AVP, especially when rats are subjected to rotatory stimulation.

However, we did not find any difference between the susceptible and insusceptible groups of rats in the SNPs of 38 sites of the V1bR gene that we investigated in the present study. It is suggested that in the 38 sites of the V1bR gene, SNPs are not involved in the difference of motion sickness susceptibility in rats.

## Conclusions

The present results suggest that AVP, through its modulatory, possibly humoral action on the VN neurones via the mediation of V1bR, may contribute to the development of motion sickness in rats; AVP gene polymorphisms may contribute to the individual difference in the responsive expression of AVP in the PVN; and higher expressions of AVP in the PVN and V1bRs in the VN may contribute to the development of motion sickness in rats after vestibular stimulation.

## Abbreviations

AVP: arginine vasopressin
cDNA: complementary DNA
CSF: cerebrospinal fluid
CTA: conditioned taste aversion
ELISA: enzyme-linked immunosorbent assay
MS: motion sickness
PBS: phosphate buffered saline
PVN: paraventricular nucleus
qRT-PCR: real-time quantitative PCR
SSS: saccharin sodium solution
V1bRs: V1b receptors
VN: vestibular nucleus
